# Podocyte Lysosome Dysfunction in Chronic Glomerular Diseases

**DOI:** 10.3390/ijms21051559

**Published:** 2020-02-25

**Authors:** Guangbi Li, Jason Kidd, Pin-Lan Li

**Affiliations:** Department of Pharmacology and Toxicology, School of Medicine, Virginia Commonwealth University, Richmond, VA 23298, USA; guangbi.li@vcuhealth.org (G.L.); jason.kidd@vcuhealth.org (J.K.)

**Keywords:** podocyte, lysosome, sphingolipids, exosome, chronic glomerular diseases

## Abstract

Podocytes are visceral epithelial cells covering the outer surface of glomerular capillaries in the kidney. Blood is filtered through the slit diaphragm of podocytes to form urine. The functional and structural integrity of podocytes is essential for the normal function of the kidney. As a membrane-bound organelle, lysosomes are responsible for the degradation of molecules via hydrolytic enzymes. In addition to its degradative properties, recent studies have revealed that lysosomes may serve as a platform mediating cellular signaling in different types of cells. In the last decade, increasing evidence has revealed that the normal function of the lysosome is important for the maintenance of podocyte homeostasis. Podocytes have no ability to proliferate under most pathological conditions; therefore, lysosome-dependent autophagic flux is critical for podocyte survival. In addition, new insights into the pathogenic role of lysosome and associated signaling in podocyte injury and chronic kidney disease have recently emerged. Targeting lysosomal functions or signaling pathways are considered potential therapeutic strategies for some chronic glomerular diseases. This review briefly summarizes current evidence demonstrating the regulation of lysosomal function and signaling mechanisms as well as the canonical and noncanonical roles of podocyte lysosome dysfunction in the development of chronic glomerular diseases and associated therapeutic strategies.

## 1. Introduction

In the early 1950s, Christian de Duve discovered “sac-like structures” that contained lytic enzymes while investigating the mechanism of action of insulin [1,2]. Following de Duve’s work, Alex Novikoff characterized the ultrastructure of these compartments, which led de Duve to rename them lysosomes [3]. In another pioneering study, Werner Strauss discovered that proteins localized in the lysosomes were fragmented by tracing the fate of radiolabeled extracellular proteins [4]. As the cell’s degradative organelle, the lysosome has gained notoriety as the “recycling center” of the cell.

Podocytes are terminally differentiated epithelial cells covering the outer surface of glomerular capillaries. They typically do not proliferate. Most glomerular diseases in which the podocyte is the target of injury are not associated with podocyte proliferation [5,6]. As the major degradative components of cells, the normal function of lysosomes is necessary to renew cellular activity and maintain the structural and functional integrity of podocytes. Over the last decade, the canonical notion of the lysosome as a simple recycling center has undergone a dramatic revolution. The mechanistic target of rapamycin complex 1 (mTORC1), the master regulator of cell growth, was found to be localized on the surface of the lysosome. As a result of this discovery, the lysosome is now recognized as a metabolic signaling hub. Recent studies have revealed the essential role of lysosome-dependent sphingolipid metabolism in glomerular disorders of genetic and non-genetic origin [7]. In this review, we focus on the lysosome as a platform for physiological and pathological signaling that controls the podocyte homeostasis and how the dysregulation of lysosome function leads to podocyte injury and glomerular diseases.

## 2. Different Types of Lysosomes

As a membrane-bound organelle in eukaryotic cells, lysosome originates from the Golgi apparatus and stays in the cytoplasm. Many acid hydrolases are responsible for intracellular digestion by lysosomes. Lysosomes are divided into two types according to their different functions: conventional lysosome and secretory lysosome. Conventional lysosome acts as a waste disposal machinery to control phagocytosis and autophagy [8,9]. Secretory lysosome can move towards and fuse to the plasma membrane, leading to the release of its contents into the extracellular space. Recently, a new type of lysosome has been found to repair the damaged plasma membrane via its fusion to plasma membrane [10,11,12,13]. This type of lysosome may also play an essential role in receptor-mediated endocytosis, leading to the recycling of receptors [14]. More recently, it has been found that exocytosis of non-secretory cells is dependent on lysosomes [11]. After lysosomes fuse to the plasma membrane, their contents are released to the extracellular space. Meanwhile, the components in lysosomal membrane are incorporated into the plasma membrane [11].

## 3. Cellular Functions Regulated by Lysosomes

**Figure 1:**
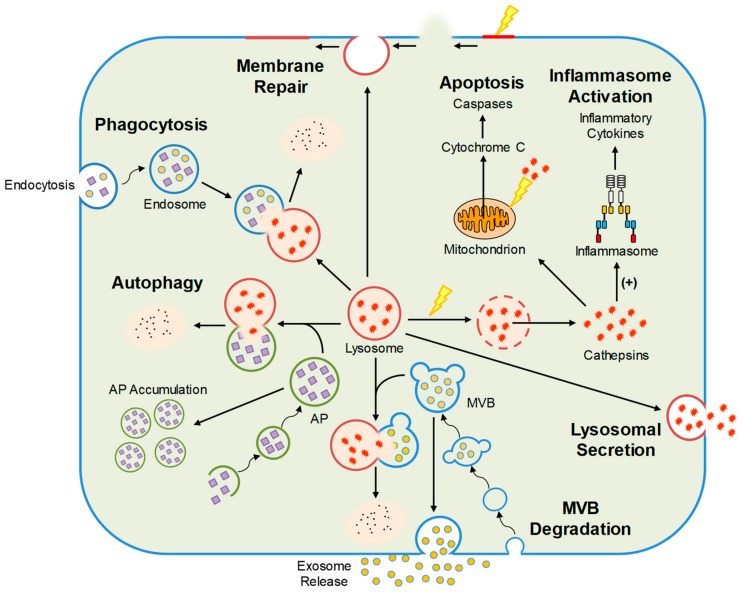
Cellular functions regulated by lysosomes including autophagy, MVB degradation, lysosomal secretion, phagocytosis, inflammasome activation, membrane repair, and apoptosis. AP, autophagosome; MVB, multivesicular body.

Intracellular destruction of endocytic, phagocytic, and autophagic materials is dependent on lysosomes which serve as the major degradative compartments with their digestive function [15,16,17]. Beyond intracellular digestion, lysosomes may mediate cellular signaling in different cells [18,19,20] such as their role in receptor recycling through an endocytic pathway [21] and as a Ca^2+^ store importantly participating in the physiological regulation of cell functions or activities in many tissues or cells [19,22,23,24,25], including podocytes [26,27,28]. In podocytes, normally functioning lysosomes are particularly important for the protection of this terminally differentiated cell type from the challenges of various danger factors [27,29,30,31]. Recent clinical and experimental studies have indicated that the deficiency or loss of lysosome function in podocytes results in proteinuria, glomerular sclerosis, and dramatically increased susceptibility to different pathological stimuli [32,33,34,35]. As shown in Figure 1, lysosomes regulate various important cellular functions. Here, we highlighted some lysosome functions which may be essential for the maintenance of podocyte homeostasis or implicated in the onset or development of podocyte injury and glomerular diseases.

### 3.1. Autophagic Flux

The acidification of lysosomal compartment facilitates the function of lysosomal hydrolases, which is dependent on H^+^-ATPases on the lysosomal membrane. The Ca^2+^ enters the lysosomal compartment by H^+^/Ca^2+^ exchange under resting conditions and exits in response to various stimuli [23,36]. The regular influx and efflux of Ca^2+^ are essential for the normal functions of lysosomes. By destroying various aged organelles within the cell such as dysfunctional mitochondria, lysosomes maintain cell function and integrity. In this regard, lysosome-dependent autophagy has been found to control the degradation of damaged or aged materials in podocytes. This autophagic flux plays an important role in the clearance of waste in podocytes under normal condition and recovery of podocytes from damage to the glomeruli upon pathological stimuli. The degradation of defective or damaged mitochondria and consequent inhibition of pro-apoptotic protein release may be the mechanisms by which lysosomes attenuate cell apoptosis [37]. Furthermore, it has been found that the differentiation and maturation of podocytes are dependent on autophagy, indicating the importance of lysosome function in maintaining the epithelial phenotype of podocytes [26,38,39]. Various important cellular functions such as receptor-mediated endocytosis, receptor recycling, and cell membrane repair [14,40,41] requires normal function of lysosomes.

The localization of the lysosome as a dynamic organelle has been reported to change in response to a variety of treatments [42]. The kinesin and dynein motors mediate the lysosome movement along the microtubule in anterograde and retrograde directions, respectively [43,44]. Moreover, intracellular pH is one of the best-known regulators of lysosomal positioning. The lysosomes redistribute from their predominantly perinuclear location toward the cell periphery in response to acidification of lysosomal compartment [45]. Furthermore, nutrients and growth factors can drive lysosomes toward the plasma membrane, whereas starvation results in lysosomes moving toward perinuclear localization [46,47]. Vacuolar-type proton pumping ATPase (V-ATPase) is a ubiquitous enzyme responsible for H^+^ transport across membranes and acidification of cellular compartments in animals [48]. The phosphate bond energy of ATP is thus converted to a proton gradient across the membrane through the mechanical rotation of subunits. The proton pumping action of lysosomal V-ATPases results in the generations of an acidic lumen and proton gradient of lysosomes, which are essential for normal functions of lysosomes [48]. A recent study in our lab has revealed that inhibition of V-ATPase activity remarkably attenuated lysosome function, leading to autophagic deficiency and podocyte dedifferentiation [26].

As a master growth regulator, mTORC1 becomes activated at the cytosolic side of lysosomes in response to nutrients, which increases the peripheral appearance of lysosomes and inhibits fusion of autophagosome and lysosome. On the contrary, starvation causes the perinuclear clustering of lysosomes and enhances autophagic flux via inhibition of mTORC1 activity [49,50]. In HeLa cells, HIV-1 counteracts metabolic and environmental stress-induced intracellular repositioning of lysosomes through enhancement of mTORC1 activity [51]. In another study, knockdown of FUT1 has been found to elevate the peripheral distribution of lysosomes by inhibition of mTORC1 activity, leading to the enhancement of autophagic flux [52]. All these previous findings demonstrate that the mTOR signaling pathway plays an important role in the regulation of lysosome positioning and trafficking. A recent study in our lab has confirmed that mTORC1 inhibition by rapamycin may increase the fusion of autophagosome and lysosome by enhancing lysosome trafficking. Rapamycin-induced enhancement of autophagic flux may be substantially blocked by nicotinamide, CD38 shRNA, and bafilomycin [28]. Furthermore, palmitate, a saturated free fatty acid, has been found to activate mTORC1, leading to the endoplasmic reticulum (ER) stress-dependent apoptosis in podocytes. Inhibition of mTORC1 activity by rapamycin or siRNA for Raptor, a component of mTORC1, ameliorated palmitate-induced ER stress and apoptosis in podocytes [53]. Together, these previous studies have demonstrated that the regulation of lysosome trafficking and autophagic flux by targeting mTORC1 is a potential therapeutic strategy against podocyte injury and glomerular diseases. However, previous studies have revealed that the basal level of mTORC1 activity is required for maintaining the normal function of podocytes. Genetic deletion of mTORC1 in podocytes may cause the disruption of autophagic flux, leading to podocyte dysfunction and proteinuria [54,55]. In addition, patients with certain renal problems may suffer progressive proteinuria by taking currently available mTORC1 inhibitors [56,57,58]. Their side effects may be due to the inhibition of the basal level of mTORC1 activity and the mTORC2-Akt pathway, which are important for maintaining the integrity of podocytes [59,60].

### 3.2. Lipid Metabolism

As a digestive organelle, the lysosome is responsible for lipid metabolism. The deprivation of nutrient may result in association of lipid droplets and autophagic membrane components [61]. The storage of triglyceride in lipid droplets is elevated after inhibition of autophagy [61]. In macrophages, the lysosome is responsible for the metabolism and transport of cholesterol and low-density lipoprotein. Under normal conditions, lysosomal acid lipase hydrolyzes cholesteryl esters to free cholesterol. This product is then actively exported out of lysosomal compartment through Niemann–Pick-type C1 protein. Several lysosomal proteins such as acid sphingomyelinase (ASM), mucolipin-1, and H^+^-ATPase regulate metabolism and transport of cholesterol in lysosomes [62].

As components of the external layer of plasma membranes, sphingolipids are transported by the endocytic vesicular flow through the early and late endosomal compartment and degraded in lysosomes [63]. In the lysosomal compartment, sphingolipids are catabolized to simple compounds, such as sphingosine, fatty acids, and sugars [63,64]. These products are exported out of the lysosomes through specific membrane proteins and then partially recycled for the biosynthesis of new sphingolipids [65,66,67]. In addition, it has been reported that many cellular activities, such as proliferation, apoptosis, differentiation, and migration, are influenced by sphingolipids [68,69]. Furthermore, sphingolipids have been found to interact with extracellular matrix, growth factor receptors, and neighboring cells [70].

### 3.3. Inflammasome Activation

As a cytosolic multiprotein platform, inflammasome assembles in response to pathogen-associated molecular patterns (PAMPs) and damage-associated molecular patterns (DAMPs) [71]. As a vital intracellular homeostatic recycling process, autophagy has been shown to negatively regulate inflammasome activation in previous studies [72,73]. Damaged organelles such as mitochondria are removed by autophagy, leading to reduced release of mitochondrial-derived DAMPs and subsequent suppression of inflammasome activation [74,75,76]. Autophagic deficiency results in ROS-producing mitochondria accumulation, leading to enhancement of NLRP3 inflammasome activation in response to ATP, monosodium urate crystals, palmitic acid, and influenza A virus [74,75,77,78]. Autophagy also regulates inflammasome activation through the lysosome-dependent degradation of inflammasome complexes. It has been reported that pharmacological inhibition of autophagy can greatly increase activation of caspase-1 and inflammatory cytokine production in response to the induction of AIM2 in monocytes [79]. Moreover, it has been found that pro-IL-1β can be sequestered into autophagosomes for degradation by lysosomes after treatment of macrophages with rapamycin, a pharmacological inducer of autophagy that inhibits mTOR [80].

### 3.4. Exosome Release

The exosomes, one of the extracellular vesicles (EVs), are released by the fusion of the multivesicular body (MVB) to plasma membrane [81]. These EVs may regulate cell-to-cell communications. After long-time challenging and debating about the characterization and classification of EVs, EVs are now classified as three distinct populations including apoptotic bodies, microvesicles, and exosomes. Among them, exosomes are the smallest EVs with approximately 50–140 nm in diameter despite no set clear cut-off size to separate them from microvesicles. Different from other EVs, exosomes are formed through the endocytic process and released from intracellular MVBs through an active process. EVs or exosomes have been extensively studied for their biogenesis and related function in cell communication and in the pathogenesis of different diseases including renal diseases [82,83,84]. In recent studies, lysosome dysfunction induced by alkaline agents and lysosomal V-ATPase inhibitor leads to increase in exosome release of various cells such as neurons, epithelial cells, and vascular cells [85,86,87]. In addition, MVBs were found to fuse with autophagosomes (APs) to form amphisomes and subsequently fuse with lysosomes to terminate MVB fate and thereby reduce exosome release [88,89,90].

As an important biomarker indicating kidney function or disease, exosomes mediate intra-renal cell-to-cell communication and contribute to the development of various renal diseases [83]. There is evidence that exosomes containing podocalyxin, a glycoconjugate on the podocyte apical surface, are increased in diabetic mice even before the onset of albuminuria [91]. In some patients with focal segmental glomerulosclerosis (FSGS) and nephrotic syndrome (NS), podocyte-derived exosomes increased in concert with albuminuria and glomerular degeneration [83,92,93,94,95,96]. Increased exosomes may serve as a signaling vesicle to trigger phenotypic changes in neighboring cells [97] and they also participate in the development of albuminuria [98].

There is evidence that the lysosome-mediated regulation can actively respond to microenvironment changes such as increased autophagosomes, MVBs or other stress signals, which occurs in podocytes and other cells. Such active regulation of lysosome function determines the disposal of different intracellular vesicles such as phagosomes, autophagosomes, and MVBs [28,99,100,101]. Previous studies have shown that regular lysosome trafficking controls the fusion of lysosomes and MVBs. The lysosomal Ca^2+^ release determines the active movement of lysosomes [25,100]. As a ubiquitously expressed protein, transient receptor potential-mucolipin-1 (TRPML1) channel is an ion channel expressed in intracellular endosomes and lysosomes [102]. The Ca^2+^ enters the lysosomal compartment by H^+^/Ca^2+^ exchange and exits through TRPML1 channels in response to endogenously produced NAADP [23,103,104,105] or other factors like PIPs (PI(3,5)P2) and ions [19,106,107].

## 4. Podocyte Lysosome Dysfunction in Chronic Glomerular Diseases

Growing evidence reveals that pathological progression of podocyte injury in glomerular diseases originates from the disarrangement of lysosome function. The resolution of podocyte injury involves alteration of autophagic flux as well as targeting lysosome-dependent sphingolipid metabolism by pharmacological intervention or enzyme replacement therapy. All the mechanisms or regulatory pathways mentioned above that control or modulate lysosome function are important for the maintenance of podocyte activity and glomerular function. If they fail to function properly, podocyte dysfunction and glomerular injury will occur. Below, we highlight the role of lysosome dysfunction and associated dysregulation in several common nephropathies associated with podocyte injury.

### 4.1. Diabetic Nephropathy

Diabetic nephropathy (DN) is the most common cause of end-stage renal disease (ESRD) worldwide [108]. Pathologic changes in the diabetic kidney including thickening of the glomerular basement membrane, mesangial expansion, proteinuria, inflammation, and fibrosis. Podocyte damage and loss contribute to the impairment of renal function in DN. In recent years, increasing evidence indicates that the disarrangement of lysosomal function is attributed to the initiation of podocyte injury and the development of ESRD during DN.

**Figure 2:**
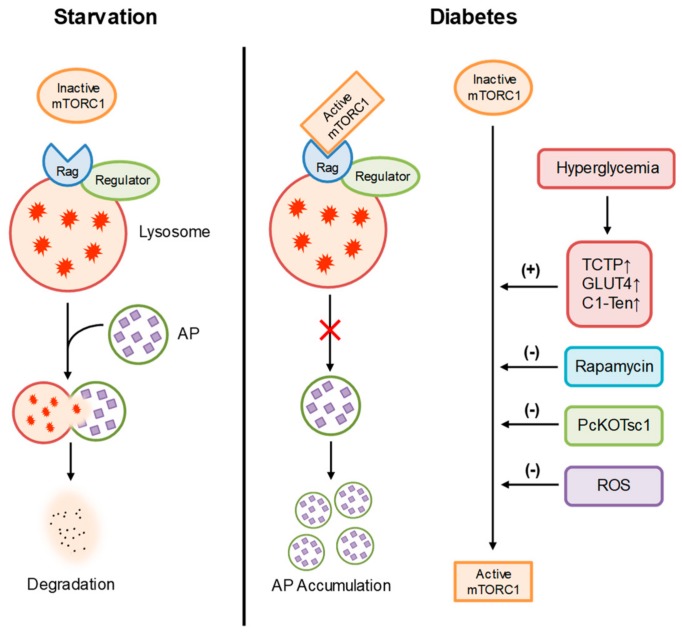
Regulation of mTORC1 in podocytes. During starvation, inactive mTORC1 has no effects on lysosome function and autophagic flux in podocytes. During diabetic nephropathy (DN), hyperglycemia upregulates TCTP, GLUT3, and C1-Ten, leading to overactivation of mTORC1 and inhibition of autophagy. As a specific mTORC1 inhibitor, rapamycin can prevent lysosome dysfunction, autophagic deficiency, and podocyte injury during diabetes. Reactive oxygen species (ROS) produced by mitochondria has also been reported to prevent mTORC1 activation in podocytes during diabetes.

Previous studies have demonstrated that autophagy is renoprotective in various renal diseases including DN [109]. As an anaphase cell with a restricted capacity in differentiation and proliferation, podocyte is very dependent on autophagy because of its self-repaired feature [110]. Strong evidence has confirmed that activation of mTOR suppresses autophagy. By this mechanism, nutrient deficiency activates autophagy by suppressing the expression of mTOR. On the contrary, mTOR is activated by both growth factors and nutrients, such as glucose and amino acids [111,112,113]. During diabetes, hyperglycemia has been found to induce the overactivation of mTOR signaling in lysosomes [114]. The use of rapamycin as a specific mTORC1 inhibitor to effectively attenuate the development of glomerular injury in the animal models of DN has established that mTOR signaling and related lysosome function have an important role in the pathophysiology of DN. In this regard, translationally controlled tumor protein (TCTP), GLUT4, and C1-Ten have been found to regulate mTOR complex 1 (mTORC1) signaling in glomeruli and the podocyte is the principal cell responsible for the regulation of mTORC1 by these proteins [115,116,117]. During diabetes, elevation of these regulators may explain for overactivation of lysosomal mTORC1 in podocytes, leading to podocyte injury and glomerular diseases. Genetic deletions of these regulators have been shown to prevent the development of diabetic nephropathy, as indicated by the amelioration of proteinuria, mesangial expansion, podocyte loss and glomerular sclerosis. Reactive oxygen species (ROS) produced by mitochondria has also been reported to prevent mTOR activation in podocytes exposed to high glucose for 24 h, leading to enhancement of lysosome-dependent autophagy which may limit the augmentation of ROS produced by damaged mitochondria [118,119]. However, chronic exposure to high glucose leads to autophagy insufficiency and subsequently causes lysosomal dysfunction, leading to podocyte apoptosis [29]. Furthermore, genetically reducing mTOR levels by eliminating the Raptor allele dramatically prevents podocyte injury and ameliorates the progression of glomerular dysfunction during diabetes [54]. In addition, podocyte-specific mTORC1 activation induced by the ablation of an upstream negative regulator (PcKOTsc1) has been reported to recapitulate many features of DN, including podocyte loss, glomerular basement membrane thickening, mesangial expansion, and proteinuria in nondiabetic young and adult mice [120]. However, it has been reported that podocyte-specific deletion of the mTORC1 gene induces proteinuria and progressive glomerulosclerosis [54]. These findings suggest that excess of both activation and inhibition of mTOR signaling may cause podocyte injury. Given the important role of mTOR signaling in autophagy, lysosome dysfunction and autophagic deficiency due to overactivation of mTOR signaling may be involved in the pathogenesis of podocyte injury and glomerular damage in patients with diabetes. Figure 2 summarizes the regulation of mTORC1 in podocytes during DN.

In addition to autophagy, lysosome-dependent lipid metabolism plays an important role in podocyte injury and glomerular diseases during DN. A previous study has confirmed that rapamycin effectively attenuates STZ-induced DN which is shown by inhibition of glomerular enlargement and proteinuria [121]. Mechanically, they have found that ceramide and sphingomyelin are both remarkably elevated in the renal cortex of rats with DN while rapamycin significantly inhibited the elevations of ceramide and sphingomyelin. These results indicate that suppression of abnormal sphingolipid metabolism contributes to the therapeutic effect of rapamycin on DN [121]. Moreover, the activation of adiponectin receptors has been found to regulate the expression of lysosomal acid ceramidase (AC) which converts ceramide to sphingosine [122]. The reductions of both adiponectin receptor and lysosomal AC were detected in diabetic mice, which were significantly attenuated by AdipoRon, an adiponectin receptor agonist. Furthermore, AdipoRon-induced enhancement of lysosomal AC activity lowered cellular ceramide levels, which may contribute to the therapeutic effects of AdipoRon [122]. More recently, a clinical study has found that the elevation of urinary ceramide is associated with DN [123]. According to results from other studies, the abnormal sphingolipid metabolism by lysosomal enzymes may contribute to the elevation of urinary ceramide in DN patients [123]. These results further confirmed that abnormality of lysosome-dependent sphingolipid metabolism might be a molecular mechanism leading to podocyte injury and glomerular damage during diabetes.

Lysosome membrane permeabilization (LMP) has been found to initiate lysosome-dependent cell death [124]. Lysosome contents including cathepsins as the main stimulator of the cell death are released during the destabilization of lysosomal membrane [125]. Recent studies have shown the important role of LMP in podocyte injury under pathological conditions such as idiopathic membranous nephropathy [27] and diabetic nephropathy (DN) [126]. As a dangerous factor elevated during DN, advanced glycation end products (AGEs) have been found to induce LMP in murine podocytes [126]. Correspondingly, cathepsins B, D, and L were released into the cytosol of these cells. The LMP initiates the development of autophagic deficiency, apoptosis, and Rac-1-dependent actin-cytoskeletal disorganization in these cells. Interestingly, exposures to both C5b-9 and AGEs have been reported to enhance the production of reactive oxygen species (ROS) [127,128], while the occurrence of LMP is frequently associated with the overproduction of ROS [129,130,131]. As an intracellular cysteine protease, cathepsin L is upregulated in podocytes and glomeruli under different pathological conditions [132,133]. A recent study targeting cathepsin L has confirmed that cathepsin L-knockout mice do not develop podocyte injury and glomerular damage during diabetes [132]. Mechanistically, CD2-associated protein (Cd2ap) and synaptopodin proteolysis are promoted by cathepsin L, leading to podocyte damage and massive foot process effacement [134]. Cathepsin abrogation is protective against a variety of external stimuli. Furthermore, cathepsin L possesses the function of cleaving dynamin, which affects the regulation of the actin cytoskeleton in podocyte foot processes [135].

**Figure 3:**
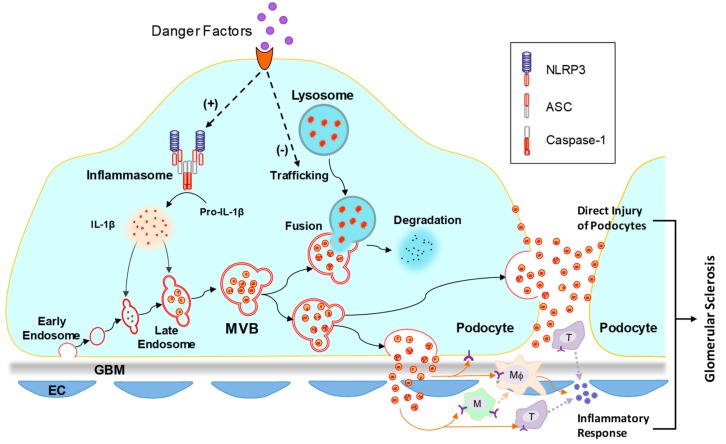
Mechanism of inflammatory exosome release in podocytes. It has been reported that in podocytes, pathological stimuli may induce inflammasome activation and lysosome dysfunction. Activated inflammasome produces proinflammatory cytokines, such as IL-1β and IL-18. Lysosome dysfunction leads to reduced MVB degradation and increased exosome release. The proinflammatory cytokines in podocytes may be released through exosome release. The released inflammatory exosomes may induce direct injury of podocytes and inflammatory response, leading to the development of glomerular sclerosis. NLRP3, nucleotide-binding oligomerization domain-like receptor containing pyrin domain 3; ASC, adaptor molecule apoptosis-associated speck-like protein containing a caspase recruitment domain; IL-1β, interleukin-1β; GBM, glomerular basement membrane; EC, endothelial cell.

Recently, it has been found that D-ribose, an overlooked risk factor in the development of type II diabetes mellitus, produces AGEs much more rapidly than D-glucose [136,137]. A recent study in our lab has demonstrated that D-ribose induces formation and activation of NLRP3 inflammasome in podocytes via AGE/RAGE signaling pathway, which may contribute to the initiation of podocyte injury and glomerular damage during diabetes [138]. After NLRP3 inflammasome is activated, sphingolipid-mediated regulation of lysosome function significantly affects the release of inflammasome-derived products into extracellular space via exosomes [139]. Both inhibition of lysosomal ASM and activation of lysosomal AC attenuated inflammatory exosome release through enhancement of lysosome-dependent degradation of MVBs [139]. In conclusion, lysosomal sphingolipid metabolism as a regulator of inflammatory exosome release may be a novel target for the development of therapeutic strategy for prevention and treatment of DN. Figure 3 summarizes the mechanism of inflammatory exosome release in podocytes.

### 4.2. Glomerular Injury during Fabry Disease

The mutation of the gene encoding alpha-galactosidase A (α-GalA), a lysosomal enzyme that hydrolyzes the terminal alpha-galactosyl moieties from glycolipids and glycoproteins, results in the systemic accumulation of globotriaoslyceramide (Gb3), which is so-called Fabry disease [140]. Elevation of urinary Gb3 is detected in patients with Fabry disease [141,142]. In the kidney, Gb3 accumulation occurred mainly within the lysosome, ER, and nuclear markers of renal cells [143], which may be reversed by enzyme replacement therapy using recombinant α-GalA [142]. In patients with Fabry disease, the pathological changes observed in podocytes included hypertrophy, lysosome enlargement, and characteristic inclusion bodies of glycolipids, which led to mesangial cell expansion [144]. A recent study has demonstrated that the intracellular Gb3 accumulates in response to the lentiviral knockdown of α-GalA in human podocytes [145]. Mechanically, the loss of mTOR kinase activity and dysregulated autophagy were associated with the intracellular Gb3 accumulation [145]. The link between α-GalA and mTOR signaling pathway has further confirmed the importance of normal Gb3 metabolism in the maintenance of podocyte homeostasis. Enzyme replacement therapy using recombinant human α-GalA has been found to attenuate Gb3 accumulation in different types of renal cells including vascular endothelial cells, vascular smooth muscle cells, mesangial cells, interstitial cells, distal tubular epithelial cells, and podocytes, which is associated with suspension of the progression of renal pathology and prevention of renal failure in patients with Fabry disease [146]. However, the clearance of Gb3 by enzyme replacement therapy in podocytes and distal tubular epithelial cells is more limited than the clearance observed in other cell types [146]. In this regard, a previous study has unveiled a strategy for specific delivery of recombinant α-GalA to podocytes. Mannose 6-phosphate/insulin-like growth II receptor, megalin, and sortilin have been identified in human podocytes [147]. It has been demonstrated these receptors possess delivery capabilities for enzyme replacement therapy. This study has identified potential pathways for potential non-carbohydrate-based drug delivery to podocytes for treatment of Fabry disease-associated podocyte injury and glomerular diseases.

### 4.3. Hyperhomocysteinemic Nephropathy

A homocysteine level that exceeds 15 μmol/L in the plasma of patients is characterized as hyperhomocysteinemia (hHcy). It has been reported that the progression of many chronic metabolic diseases including hypertension, peripheral vascular disease, Alzheimer’s disease, diabetes and atherosclerosis is attributed to elevated homocysteine (Hcy) [148]. Accumulation of Hcy or hHcy in the blood induces pathological alterations in the glomeruli including extracellular matrix accumulation and podocyte injury. The inability of this toxic compound to be properly cleared or degraded from the body eventually leads to compromised renal function and glomerulosclerosis [149,150]. Although it remains unclear how hHcy causes cellular injury and sclerotic changes in many organs and tissues, some studies have revealed that ceramide production is upregulated during hHcy, suggesting this sphingolipid may play a crucial role in these processes [151,152,153]. In this regard, a recent study in our lab has demonstrated that hHcy-induced NADPH oxidase-dependent superoxide production may be attributed to overexpression of lysosomal ASM and consequent ceramide accumulation in the glomeruli of hHcy mice [154]. A further study has confirmed that hHcy-induced glomerular ceramide accumulation occurs mainly in the podocytes [155]. In vivo evidence shows that ASM gene deletion blocks glomerular superoxide production and podocyte injury in mice with hHcy. Pharmacological inhibition of ASM by amitriptyline has been found to prevent ceramide accumulation, superoxide production, and cellular injury in cultured murine podocytes [155]. However, it remains unclear how the overproduction of ceramide by ASM leads to podocyte injury and glomerular diseases during hHcy.

Since superoxide production can induce inflammasome activation, we investigated whether inflammasome is involved in podocyte injury during hHcy. Strong evidence has confirmed that NLRP3 inflammasome formation and activation are important molecular mechanisms triggering podocyte injury and ultimately resulting in glomerular sclerosis during hHcy [156,157]. Moreover, endogenously produced reactive oxygen species has been found to contribute to the activation of NLRP3 inflammasome in podocytes during hHcy [158]. Recently, we have confirmed that the overproduction of ceramide by lysosomal ASM induces NLRP3 inflammasome activation in podocytes, leading to proteinuria and glomerulosclerosis [159]. The assembly and activation of the NLRP3 inflammasome may be attributed to superoxide production in glomeruli during ceramide accumulation. These results indicate that an imbalance of lysosome-dependent sphingolipid metabolism may be the molecular mechanism initiating NLRP3 inflammasome activation in podocytes, leading to podocyte injury and glomerular sclerosis during hHcy.

### 4.4. Obesity-Induced Podocyte Injury and Glomerular Disease

Previous studies have revealed that obesity is a risk factor of chronic kidney disease (CKD) and end-stage renal disease (ESRD) [160,161]. Adipose tissue, especially visceral fat, generates bioactive substances which contribute to pathological changes of renal hemodynamics and structure, leading to glomerular injury [162]. These bioactive substances derived from adipose tissue include the adipokines visfatin, leptin, and adiponectin as well as various cytokines such as tumor necrosis factor-α (TNF-α), resistin, and interleukin-6 (IL-6) [162]. As an anti-inflammatory adipokine, adiponectin inhibits pro-inflammatory cytokine release, enhances anti-inflammatory cytokine release, and restores intracellular ATP levels [163,164,165]. It has been reported that adiponectin can inhibit the development of various obesity-related diseases [163,166,167]. Recently, we have demonstrated that adiponectin inhibits pannexin-1 (Panx1) channel activity in podocytes via enhancement of lysosomal AC activity [168]. Since the Panx1 channel mediates ATP release, inhibition of the Panx1 channel may contribute to the anti-inflammatory property of adiponectin. Furthermore, the expression of AC has been found to be upregulated by activation of adiponectin receptors [122]. Therefore, hypoadiponectinemia may lead to enhancement of Panx1 channel activity during obesity, leading to increased ATP release, inflammasome activation, and consequent inflammatory response in local tissues such as glomeruli. Moreover, the high-fat diet (HFD) has been found to significantly increase glomerular ceramide production, NADPH oxidase-dependent superoxide production, and NLRP3 inflammasome formation in glomeruli of wild type mice [159]. The activation of NLRP3 inflammasome mainly occurs in podocytes. However, the deletion of the ASM gene totally blocked NLRP3 inflammasome activation and glomerulosclerosis during obesity [159]. These results indicate that the normal function of lysosomal ASM is essential for the maintenance of podocyte homeostasis. The imbalance of lysosome-dependent sphingolipid metabolism may induce NLRP3 inflammasome activation, leading to podocyte injury and glomerular sclerosis during obesity.

**Figure 4:**
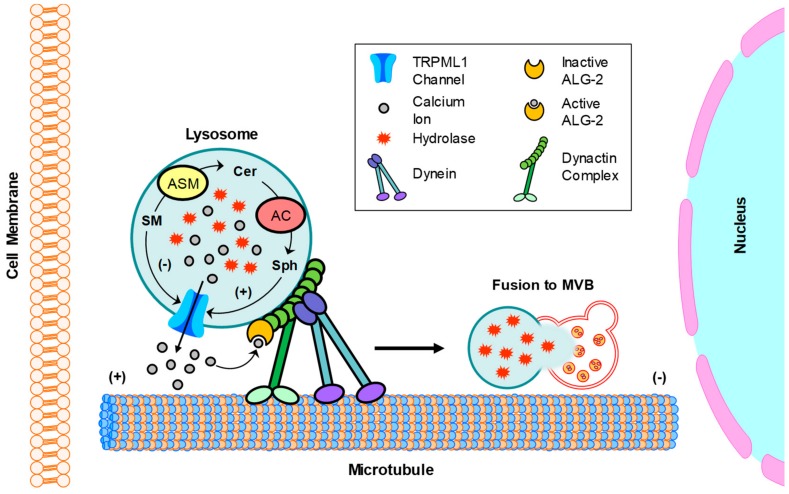
Regulation of lysosome trafficking by sphingolipids in podocytes. Dynein-mediated retrograde transport of lysosomes promotes their fusion with MVB. This transport is dependent on the TRPML1 channel-mediated Ca^2+^ release. In the lysosome, ASM converts SM into CER and AC converts CER to Sph. These sphingolipids, SM, Cer, and Sph had different effects on TRPML1 channel activity in podocytes, with inhibition by SM, no effect from Cer, but enhancement by Sph. SM, sphingomyelin; Cer; ceramide; Sph, sphingosine.

Recently, a clinical study has confirmed that urinary excretion of sphingolipids occurs in adolescents with severe obesity despite the absence of microalbuminuria. Therefore, urinary sphingolipids may be used as a parameter to detect early glomerular injury in adolescents with severe obesity [169]. The elevated urinary sphingolipids include ceramides, sphingomyelin, and glycosphingolipids. Given the recently discovered role of ceramide-enriched exosome as a passenger of danger signal and a stimulator of further damages [170,171,172], it is possible that the exosome plays a significant role in the development of glomerular diseases during obesity. In this regard, a recent study in our lab has revealed that TRPML1 channel-mediated Ca^2+^ release controls lysosome trafficking and lysosome-MVB interaction in podocytes [173]. Blockade of TRPML1 channel due to lysosomal AC dysfunction inhibits the fusion of lysosomes and MVBs, leading to enhanced exosome release from podocytes. Under pathological conditions, deficiency of lysosomal AC function may inhibit TRPML1 channel activity, leading to enhancement of podocyte-derived exosome release and consequent podocyte injury and glomerular damage. Figure 4 summarizes the regulation of lysosome trafficking by sphingolipids in podocytes.

Although autophagy has been confirmed to be essential for the maintenance of podocyte differentiation, its role in obesity-induced podocyte injury may be different. Recently, it has been found that inhibition of GLUT4 translocation to the plasma membrane induces excessive autophagy in podocytes, which contributes to the development of obesity-related glomerulopathy [174]. The glucagon-like peptide-1 analog inhibits excess of autophagy in podocytes to prevent obesity-related glomerulopathy [174]. On the contrary, rapamycin, an autophagy inducer, was found to worsen podocyte injury induced by palmitic acid [174]. These results indicate that both inhibition and excess of lysosome-dependent autophagy can cause podocyte injury and glomerular diseases under pathological conditions.

### 4.5. APOL1-Associated Nephropathy

As a minor apoprotein component of high-density lipoprotein, apolipoprotein L1 (APOL1) is expressed in different tissues, such as kidney, liver, pancreas, and brain [175,176]. Recently, increasing evidence has indicated that a major disparity in renal health is strongly associated with two coding sequence variants in APOL1 [177,178,179,180]. Compared with European Americans, African Americans have higher possibility of suffering progressive nephropathy, including FSGS, human immunodeficiency virus (HIV)-associated nephropathy (HIVAN), and hypertension-associated ESRD [181,182]. Many African Americans possess risk variants of APOL1. On the contrary, coding sequence variants of APOL1 occur infrequently in European Americans [181,183,184]. This remarkable population disparity has confirmed that abnormality of APOL1 contributes to the development of progressive nephropathy.

A clinical study has shown that patients with FSGS and HIVAN have lower expression of APOL1 in podocytes compared to other kinds of cells [185]. In a recent study, enhanced lysosomal swelling and necrosis have been found in human podocytes expressing APOL1 variants [186]. The enhanced lysosomal swelling may be due to increased lysosomal membrane permeability in podocytes with APOL1 variants. As a secreted protein, APOL1 can induce lysosomal membrane depolarization and continuous chloride influx [187]. Correspondingly, inhibition of chloride channel and blockade of interaction between APOL1 and lysosome have been found to attenuate lysosomal swelling and podocyte injury due to APOL1 variants [186]. In this regard, an in vivo study has shown that transgenic expression of human APOL1 risk variants in podocytes induces kidney disease in mice [188]. Podocyte foot process effacement, albuminuria, and glomerular sclerosis were found in mice with podocyte-specific inducible expression of the APOL1 risk variants. Mechanically, podocyte-specific inducible expression of the APOL1 risk variants may inhibit autophagic flux and induce inflammatory cell death, leading to autophagosome accumulation and podocyte loss [188]. More recently, it has been found that the interaction between APOL1 and microRNA-193a is involved in high glucose-induced podocyte dedifferentiation [189]. High glucose induced both reduction of APOL1 and upregulation of microRNA-193a which contributed to podocyte dedifferentiation. Moreover, APOL1 siRNA upregulated microRNA-193a and overexpression of microRNA-193a downregulated APOL1 in podocytes [189]. A further study has demonstrated that disruption of APOL1-microRNA-193a axis induces podocyte dedifferentiation through blockade of autophagic flux [190]. The expression of APOL1 risk variants has also been found to dedifferentiate podocytes through autophagic deficiency. In conclusion, APOL1 as a regulator of lysosome function may be a novel target for the development of therapeutic strategy against podocyte injury and glomerular diseases.

## 5. Therapeutic Potential of Chronic Glomerular Diseases by Restoring Lysosome Function

The prolonged clinical silence in chronic glomerular diseases leads to irreversible damage including accumulation of extracellular matrix proteins, proteinuria, glomerular hypertrophy, glomerular basement membrane thickening, mesangial expansion and glomerular fibrosis. Early detection of these pathological changes is necessary to facilitate therapies that can improve clinical outcomes. Management of risk factors such as hypertension, hyperglycemia, and albuminuria is essential for slowing progression to ESRD. Although beneficial effects have been confirmed in the usage of traditional therapeutics such as angiotensin-converting enzyme inhibitors, angiotensin receptor blockers, mineralocorticoid receptor antagonists, and statins [191], the morbidity and mortality of patients with CKD remain high. The identification of new therapeutic targets and the development of new strategies for the treatment of chronic glomerular diseases are imperative and there is a need to search for new pathways.

The abnormality of mTOR pathway signaling has been found to participate in all the key steps of DN progression, including damage and loss of podocytes, an early event in DN that further causes glomerular sclerosis. Increasing evidence has confirmed the efficacy of mTOR inhibitors in treating DN [192,193,194,195,196], although they may cause hyperglycemia due to the combination of impaired insulin secretion and insulin resistance [197]. Rapamycin is a well-known mTOR inhibitor, but its limited bioavailability led to the development of semisynthetic analogs, named rapalogues, which possesses improved pharmacokinetic properties and superior aqueous solubility. There are clinical evidences showing the serious disadvantage of rapalogues in terms of its desired therapeutic effects, and its cytostatic effect may partially inhibit its efficacy [198]. Furthermore, mTORC1 is the only target of rapamycin and rapalogues. The long-term treatments with these molecules may initiate aberrant feedback loops in mTOR network, leading to abnormal activation of compensatory pro-survival signaling pathways. The therapeutic effects of these treatments on glomerular diseases during diabetes can be severely compromised by these side effects. Another drawback of this approach was the ablation of raptor expression in podocytes and consequent development of proteinuria, which is consistent with the side effects of rapamycin in both animal models and humans [199,200,201,202,203]. In conclusion, increasing evidences have confirmed the significant contribution of mTOR activation in the development of DN by acting on different types of renal cells. However, this theoretically attractive therapeutic target is still lack of clinical trials using mTOR inhibitors against DN. The side effects of mTOR inhibitors in transplanted patients, such as dyslipidemia, hyperglycemia, and insulin resistance, may explain the scarceness of progression in clinical studies. Notably, pharmacological inhibition of mTORC1 with rapamycin is the only interfering method in most of the previous studies on the role of mTOR in the development of DN. A recent study has indicated that activation of mTORC2 also contributes to podocyte apoptosis and albuminuria in diabetic mice, which provides a novel therapeutic strategy for further exploration [175]. More recently, rapamycin has been found to regulate microRNA expression in kidney [204]. Furthermore, inhibition of microRNA-21 has been shown to inhibit abnormal activation of the PI3K/Akt/mTOR pathway and thereby enhance autophagic flux, leading to attenuation of high glucose-induced podocyte injury [205]. Correspondingly, it has been demonstrated that inhibition of microRNA-217 can block high glucose-induced podocyte injury by restoring autophagic flux [206]. These findings indicate that targeting microRNA may be a novel approach to regulate mTORC1 activity. However, it remains unknown whether long-term modification of microRNA produces side effects during treatment of DN. More experiments need to be performed to enhance our understanding of this therapeutic strategy.

EVs such as exosomes are found in almost all biofluids and the cargo of EVs change with disease states. This property positions EV as a potential source for the discovery of novel biomarkers of different diseases. In this regard, the urinary exosome has been reported to be a novel biomarker for renal disease [207,208,209]. Moreover, the stability and enrichment of miRNA in the exosome make it a promising candidate as a biomarker for different glomerular diseases [210,211,212,213,214,215]. However, recent studies have indicated that exosomes derived from glomerular cells such as podocytes may not be regarded only as an indicator of glomerular status. Furthermore, the biogenesis, degradation, and release of exosomes could be a novel target for the development of therapies against CKD. For example, D-ribose has been shown to induce NLRP3 inflammasome activation and inflammatory exosome release in podocytes both in vitro and in vivo [139]. In addition, TRPML1 channel-mediated Ca^2+^ release has been demonstrated to control lysosome trafficking, MVB degradation, and exosome release in murine podocytes [173]. Convincing evidence has shown that an imbalance of sphingolipid metabolism in lysosomes may block lysosome function and thereby results in the enhancement of exosome release. These released exosomes may induce or worsen the pathological changes in podocytes and glomeruli. Both inhibitions of lysosomal ASM by amitriptyline and activation of lysosomal AC by genistein have been confirmed to attenuate ceramide accumulation and podocyte injury [139,173]. However, there are still many steps before the therapeutic strategy of targeting exosome release being tested in preclinical studies. To our knowledge, amitriptyline has been demonstrated to act as an agonist for TrkA and TrkB receptors [216]. In addition, amitriptyline is a PARP1 inhibitor [217]. Furthermore, Amitriptyline acts as an antagonist or inverse agonist of serotonin receptors, α1-adrenergic receptor, histamine receptors, and muscarinic acetylcholine receptors, and as an agonist of sigma σ1 receptor [218,219,220]. Many side effects have been observed while taking amitriptyline. Therefore, new functional inhibitors of ASM with high efficacy and selectivity are required for clinical usage. As a well-known medicine for the treatment of mental illnesses, amitriptyline may affect the central nervous system if taking it for a long time. To overcome this obstacle, podocyte-specific aptamer-mediated targeted drug delivery may be a potential solution.

It is expected that more therapeutic strategies will be forthcoming, which include the use of ASM inhibitor, AC inducer, AdipoR agonist, TRPML1 channel agonist, mTOR inhibitor, lysosome stabilizer, and cathepsin inhibitor. These potential therapeutics may target different components of lysosome function, which may be selected for the use in the prevention or treatment of ESRD and associated glomerular diseases.

## 6. Concluding Remarks

This review briefly summarizes current evidence about molecular mechanisms by which the lysosome affects podocyte function and integrity under pathological conditions, including the involvement of mTORC1, microRNA, APOL1, cathepsin, α-GalA, ASM, AC, and TRPML1 channel. All these studies have provided innovative insights into ways to prevent aberrant lysosome function with the goal of preventing the development of podocyte damage and progressive renal insufficiency. The crosstalk between lysosomes and podocytes may be activated by pathological stimuli such as hyperglycemia, hHcy, obesity, cytokines, adipokines, and genetic defects. Therefore, lysosomes have been implicated in the development of a variety of renal diseases due to their induction of podocyte dysfunction and injury, which results in glomerulosclerosis, ultimately leading to ESRD. A deeper investigation is of the utmost importance to understand how these various signaling pathways interact to regulate lysosome function in podocytes, which may promote the development of more effective therapies for the prevention or treatment of glomerular diseases and consequent ESRD.
